# Investigating the Impact of Robotic Milling Parameters on the Surface Roughness of Al-Alloy Fabricated by Wire Arc Additive Manufacturing

**DOI:** 10.3390/ma17194845

**Published:** 2024-09-30

**Authors:** Zhaoyang Yan, Xikang Ren, Hongyan Zhao, Shujun Chen

**Affiliations:** 1 College of Mechanical & Energy Engineering, Beijing University of Technology, Beijing 100124, China; yanzy@bjut.edu.cn (Z.Y.); renxikang@gmail.bjut.edu.cn (X.R.); sjche@bjut.edu.cn (S.C.); 2 The State Key Laboratory of Mechanical Transmissions, Chongqing University, Chongqing 400044, China

**Keywords:** additive manufacturing, robotic milling, additive–subtractive hybrid manufacturing, aluminum alloy, surface roughness

## Abstract

This paper takes the single-wall wall manufactured by wire arc additive manufacturing (WAAM) as the research object and compares it with the as-cast aluminum alloy with the same series. By using feed rate, cutting depth, spindle speed, etc., as single or compound parameters, the machinability of the sample is analyzed. The results indicate that the influence of varying parameters on the as-deposited aluminum alloy follows the order of feed rate > cutting depth > spindle speed. As the feed rate increases, the surface roughness initially decreases and then increases, with the optimal surface quality achieved at 12 mm/s (with a surface roughness of 2.013 μm). Different from the as-deposited alloy, the influence of the parameters on the as-cast alloys follows the order of spindle speed > cutting depth > feed rate. The experiments reveal that, for both as-deposited and as-cast states, the trends of the impact of cutting depth and spindle speed on surface quality are consistent. However, at low feed rates (2–12 mm/s), for as-deposited states, the surface quality of as-deposited samples becomes smoother as the feed rate increases (contrary to common knowledge). This result can be attributed to the elevated milling temperature, which softens the material, making it easier to remove and reducing the surface roughness.

## 1. Introduction

With the rapid advancement of modern industry, large-sized and complex structural components are increasingly used in aviation, aerospace, energy, transportation, and other fields. These components often feature unique designs, intricate internal structures, and stringent performance requirements, posing significant challenges to traditional manufacturing processes [1]. Recently, the development of additive manufacturing (AM) technology has facilitated the production of large-sized aluminum alloy components, offering advantages such as a low cost, short processing cycles, and high material utilization rates [1,2,3,4,5]. AM is widely regarded as a pivotal driver of the Fourth Industrial Revolution and a predominant method for producing high-performance parts [6,7]. During wire arc additive manufacturing (WAAM), issues such as the large heating area of the arc column and energy dispersion lead to rough surfaces of the samples, requiring secondary machining and making it difficult to manufacture complex structures [8,9]. As a result, parts produced via WAAM lack precision and require further machining or milling before use. To address the demands for a certain level of production capacity and processing accuracy, hybrid manufacturing combining material addition and removal technology has emerged as a necessary means for the small-batch or single-piece production of such parts [10].

Hybrid manufacturing refers to a new type of manufacturing process that combines two or more existing manufacturing technologies. Its purpose is to achieve complementary advantages of multiple manufacturing processes in order to meet the production demands of high efficiency, high quality, and diversification in modern manufacturing industries [11]. The extant manufacturing processes are categorized into three types: AM (Δm > 0), subtractive manufacturing (Δm < 0), and equal material processing (Δm = 0). Multi-robot collaborative hybrid manufacturing, which combines WAAM and robot milling, is a novel manufacturing approach. WAAM constructs parts by adding material layer upon layer, offering high design freedom and material utilization. However, during the construction process, WAAM may introduce forming defects such as overfilling and underfilling, which can significantly impact the quality of the finished parts. Robotic milling, on the other hand, shapes the desired form by removing excess material, characterized by high precision and superior surface quality [12].

Hybrid additive–subtractive manufacturing (HASM) technology amalgamates the advantages of both additive and subtractive manufacturing. Karunakaran et al. developed a hybrid manufacturing system based on arc welding and milling, integrating a pulsed gas metal arc welding machine into a three-axis CNC (Computer numerical control machine tools) machine. After each layer is deposited, only the top surface is milled until near-net shaping is complete, followed by the final peripheral milling to ensure dimensional accuracy [13]. Jeng et al. introduced a selective laser cladding and milling hybrid process, comprising a CO_2_ laser, a coaxial powder delivery system, and a four-axis linked machining and control system. This approach involves planar milling after every two or three layers, balancing formative efficiency and quality [14]. Zhang Haiou and associates have advanced a hybrid manufacturing technique based on plasma deposition molding and CNC milling, utilizing plasma beam molding, which offers advantages like a high heat input, small beam radius, and concentrated energy, thus enabling faster accumulation and reduced costs. All the aforementioned studies were achieved through system integration, which consolidated both additive and subtractive manufacturing processes into a single device. Initially, WAAM was employed to swiftly construct an approximate form of the component. Subtractive manufacturing was utilized to perform precise finishing on the component, ensuring that it met the required dimensional accuracy and surface quality. This technology not only significantly enhanced manufacturing efficiency but also effectively reduced production costs, making it particularly suitable for the small-batch or single-piece production of large-sized, structurally complex components [15].

HASM, by incorporating subtractive processing into traditional AM, effectively enhances the dimensional accuracy and surface quality of components produced solely through additive methods, enabling complex-shaped parts to meet final application requirements. However, current research on subtractive processes in HASM remains limited, with traditional milling techniques of as-cast still being utilized. HASM aims to address the deficiencies of AM, improve production efficiency, reduce costs, enhance component performance, and drive the development of the manufacturing industry. Given that the manufacturing methods and internal microstructures of traditional as-cast are fundamentally different from those of additively manufactured components, there are significant differences in their milling processability, making it unfeasible to directly apply traditional milling processes to HASM. Therefore, it is imperative to investigate the milling processability of additively manufactured samples, providing technical and theoretical foundations for HASM [16,17].

To address the milling process of additively manufactured samples in HASM, it is necessary to analyze the surface roughness and mechanical properties of both traditionally as-cast and as-deposition samples after milling, revealing the differences in milling processes between these two material states within the same material series. Based on the characteristics, research focused on developing milling tools, milling parameters, and processing strategies suitable for HASM. Through experimental validation and analysis, an applicable milling process plan was proposed to provide technical support for the application of HASM in the small-batch or single-piece production of large-sized, complex structural components.

## 2. Materials and Methods

### 2.1. Experimental Setup

**Figure 1:**
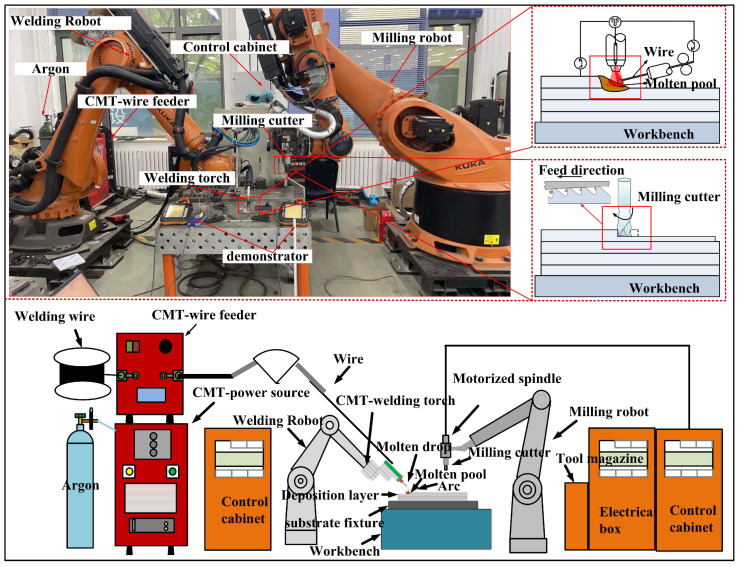
Schematic diagram of the experimental setup.

**Figure 2:**
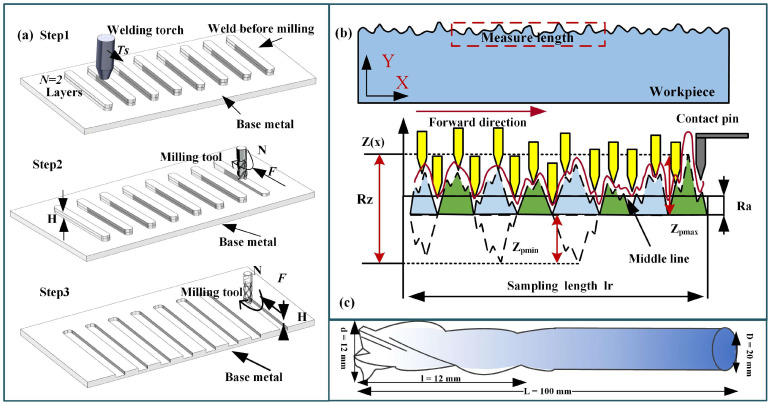
Processing process. (**a**) principle of experimental process, (**b**) roughness measurement principle, (**c**) tool specifications.

The experimental setup involved a KUKA KR210 robot (KUKA AG, Augsburg, Bavaria, Germany) and a Fronius CMT Advanced welding machine (Hong chuang Technology, Tangshan, Beijing). Subtractive manufacturing employed a KUKA KR500 robot (KUKA AG, Augsburg, Bavaria, Germany) with an ES779 high-speed milling head (Senge Machinery Co., LTD., Beijing, China), capable of reaching 22,000 r/min. In this study, a carbide three-blade cutter was selected, with a total length of 100 mm, a blade length of 45 mm, a diameter of 12 mm, and a helix angle of 55 degrees. The cutter rotated in a clockwise direction, and no coolants or lubricants were used during the experiment to maintain its purity. Figure 1 illustrates the process of HASM, while Figure 2 depicts the milling process and surface roughness measurement principles.

All single-wall structures were fabricated using the same parameters when performing WAAM on 5052 aluminum plates. Subsequently, the top layer of the as-deposited material was milled using high-speed robot milling (KUKA AG, Augsburg, Bavaria, Germany). To comparatively analyze the differences in milling processability between the as-deposited and as-cast aluminum alloy, the same series of as-cast aluminum alloy was also milled, and the surface quality was measured. To accurately measure and analyze the roughness of the processed surface, a Mitutoyo MR200 surface roughness tester (Mattel Technology, Beijing, China) was used. To further investigate the texture of the milled surface and the variation patterns of surface roughness, a high-precision laser confocal microscope (Olympus, Shinjuku, Japan) was used. To measure the hardness of the sample, the HBE-3000 series Brinell hardness tester (Lianer, Shanghai, China) was used.

### 2.2. Experimental Procedure

#### 2.2.1. The Selection of the Direction for Robot Milling

To systematically investigate the impact of milling parameters on the quality of the processed surface and eliminate potential errors caused by the milling direction of the robot, a crucial step was designed in this study. Initially, the milling depth, spindle speed, and feed rate were fixed at 0.5 mm, 4500 r/min, and 7.5 mm/s, respectively, while the milling direction was treated as a variable parameter. The robot was programmed to mill along four directions: positive X, negative X, positive Y, and negative Y, with each direction repeated three times to enhance data reliability. A roughness tester was used to precisely measure the processed surface, with a measurement length set at 12.5 mm and a Z-axis resolution of 160 μm. To ensure accuracy, each set of experimental data was collected three times and averaged. The experimental results and the processed surfaces from different milling directions are presented in Table 1. It is worth noting that the same batch of cutting tools was used throughout the experiments, and the wear of the tools was inspected before each experiment to ensure consistent experimental conditions.

After a thorough analysis of the processed surfaces, it was found that the feed direction of the robot had a significant impact on the quality of the processed surface. When milling in the Y direction, the surface quality of the part was better than that in the X direction, and the data variation in the Y direction was less pronounced compared to the X direction. This phenomenon was primarily due to the fact that the feed direction during milling significantly influenced the values of the diagonal elements of the robot’s Cartesian space stiffness matrix, meaning that the values of the robot’s end stiffness matrix were closely related to changes in its Jacobian matrix [18]. Due to the stiffness of the robot body, the Jacobian matrix might become ineffective, subsequently affecting changes in the robot’s posture. Therefore, to reduce overall experimental error, all milling directions in subsequent experiments in this paper were selected along the Y-axis.

#### 2.2.2. Experimental Design

To comparatively analyze the milling machinability of as-cast and as-deposition materials, this study designed both single-factor and multi-factor interactive experiments. In the single-factor experiments, this study focused on examining three key influencing factors: milling depth, spindle speed, and feed rate. This study detailed the selection range for each parameter and analyzed the mechanism by which each parameter affected the surface morphology. Considering that the normal operating speed of the spindle is approximately 80% of its maximum speed, and taking into account actual working conditions, the maximum spindle speed was set at 6000 r/min, the maximum feed rate at 18 mm/s, and the maximum milling depth at 1.5 mm in this experiment.

For the multi-factor interactive experiments, this study adopted the response surface method and chose a central composite rotatable design (CCRD) as the regression model. The CCRD has been proven to be an efficient experimental design method that requires relatively few experiments while maintaining accuracy. When applying a CCRD, the following procedures were followed: (1) identification of the major factors affecting the response; (2) determination of the upper and lower bounds; (3) generation of the experimental design matrix; (4) conducting the experiments based on the design matrix; (5) development of the regression model; (6) verification of the adequacy of the established model through analysis of variance (ANOVA) [19].

Before the experiments began, the ranges of various process parameters were determined and coded, as shown in Table 2. In the table, ★ represents level one, ◆ represents level two, and ▲ represents level three. This is a three-factor, three-level experimental design. After each set of experiments, a roughness tester was used to measure the surface roughness, with six points measured per experiment and an average value taken. Through regression analysis of the experimental data, a quadratic regression equation for the response variable (Ra) and input variables (y_1_, y_2_, y_3_) was obtained, and its goodness of fit, regression coefficients, and other aspects were tested. The specific regression equation is as follows:

(1) $$Ra=b_{0}+b_{1}y_{1}+b_{2}y_{2}+b_{3}y_{3}+b_{12}y_{1}y_{2}+b_{13}y_{1}y_{3}+b_{23}y_{2}y_{3}+b_{11}y_{1}^{2}+b_{22}y_{2}^{2}+b_{33}y_{3}^{2}$$

A total of 17 experiments were required for this study, including 6 factorial points, 4 star points, and 4 center points. The obtained experimental design matrix is shown in Table 3.

## 3. Results

**Figure 3:**
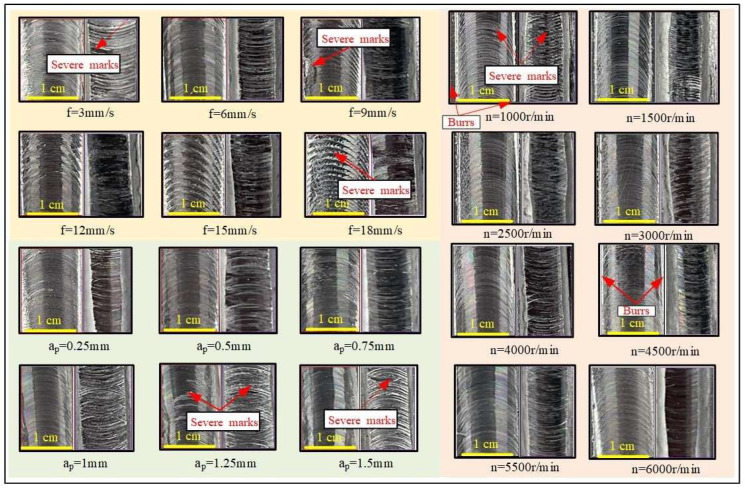
Single-factor experiments. The yellow background indicates the shape of the sample changing the feed speed, the pink background—indicates the shape of the sample changing the spindle speed, green background—indicates the shape of the sample changing the cutting depth.

This section explores the impact of different parameters on the surface quality during robotic milling. Surface morphology and surface roughness are two key indicators for assessing surface quality, with surface morphology serving for qualitative evaluation and surface roughness providing quantitative data. To clarify the specific effects of various parameters on surface morphology, this study employed an optical microscope to observe the surface morphology of samples in each group. When selecting the processing area, this study specifically chose the middle part as the sampling area, set the cut-off length and sampling length to 0.8 mm and 12.5 mm, respectively, and controlled the sliding speed of the probe at 1 mm/s. During the measurement process, to reduce random errors, three points were non-uniformly selected along the feed direction on the measured surface for measurement, and the average value of these three points was taken as the final data. Figure 3 presents the measured surface roughness results, which reveal that, as the feed rate increases, the surface morphology of the as-cast samples exhibits a notable deterioration trend, while the as-deposited samples are relatively less affected by the feed rate. With an increase in spindle speed, the surface morphology of both types of samples improves significantly. However, as the milling depth increases, the surface morphology of both deteriorates noticeably. To further analyze the impact of parameters on surface roughness, visualization processing is performed based on the results, yielding specific insights into the effects of each factor at different levels on surface roughness.

### 3.1. Spindle Speed

**Figure 4:**
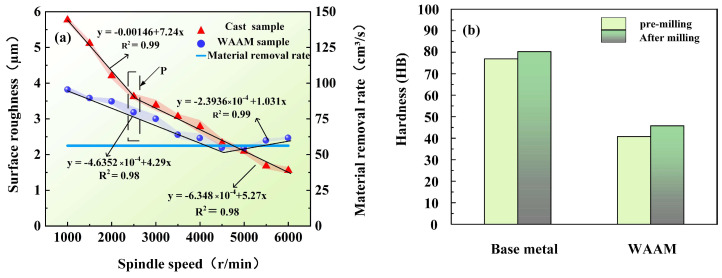
Spindle speed experiment, (**a**) the relationship between spindle speed and surface roughness, (**b**) hardness of point P in (**a**).

Figure 4a illustrates the pattern between spindle speed and surface roughness. The experimental results indicate that the surface roughness of the as-cast samples decreases as the spindle speed increases, with a particularly rapid decrease within the range of 1000 to 2500 rpm; after 2500 rpm, the rate of decrease gradually slows down. For the as-deposited samples, the surface roughness exhibits a trend of decreasing first and then increasing as the spindle speed increases. Within the range of roughness decrease, the declining trend of the additive manufactured samples is gentler compared to that of the cast samples. The variation range is primarily between 2.142 and 3.750 μm, and the optimal surface quality is achieved at a spindle speed of 4750 rpm.

To quantify this relationship, further linear modeling of the experimental data was conducted to determine the linear function between different spindle speeds and surface roughness. Specifically, hardness analysis was performed on the samples at the inflection point P in the experimental parameters (spindle speed of 4750 rpm, feed rate of 7.5 mm/s, and cutting depth of 0.75 mm). The experimental results reveal that the hardness of the as-cast samples before milling is 76.910 HB, which increases to 80.332 HB after milling, whereas the hardness of the as-deposited samples before milling is 40.751 HB, which increases to 45.252 HB after milling. The hardness of the as-deposited samples is 46% lower than that of the as-cast samples. The milling process produces a certain degree of surface hardening effect on both, as shown in Figure 4b.

**Figure 5:**
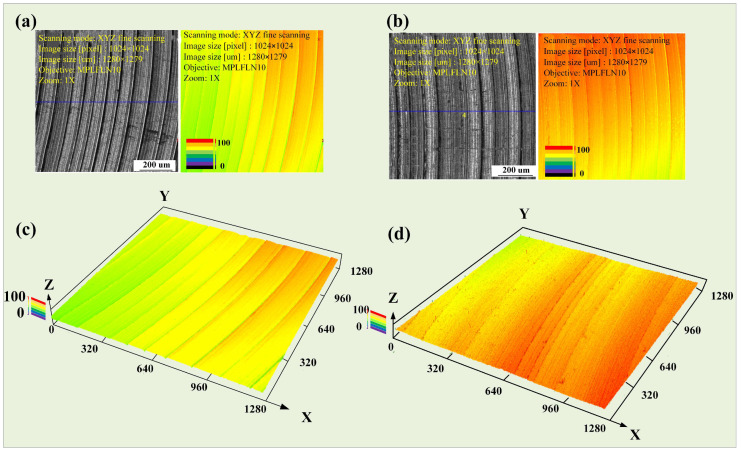
Spindle speed experiment, (**a**) surface of as-cast sample, (**b**) surface of as-deposited sample, (**c**) 3D morphology of as-cast sample, (**d**) 3D morphology of as-deposited sample.

**Figure 6:**
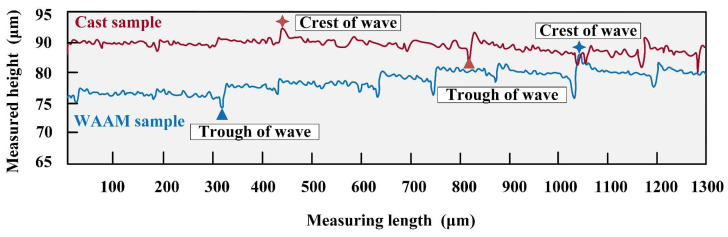
Surface contours of P point.

To clarify the differences between as-cast and as-deposited samples after milling, a precise scan of the processed surface morphology under the parameters at point P was conducted using LEXT, OLS4100 (Olympus, Shinjuku, Japan). The surface of the as-deposited sample was relatively uneven, while the surface of the as-cast sample exhibited a stepwise increasing trend. The tool marks were particularly pronounced after one complete rotation of the tool. In comparison, the height fluctuations on the surface of the as-deposited sample were larger. This difference was visually represented in the two-dimensional surface maps (Figure 5a,b) and three-dimensional topography maps (Figure 5c,d). Further measurements of the surface contours on the same measurement plane revealed that the difference in height between the as-deposited and as-cast samples reached 10.125 μm at the same height. The difference between the peak and valley of the as-cast sample was 7.503 μm, while that of the as-deposited sample was 13.512 μm, indicating greater surface fluctuations on the as-deposited sample; the results are shown in Figure 6.

### 3.2. Feed Rate

**Figure 7:**
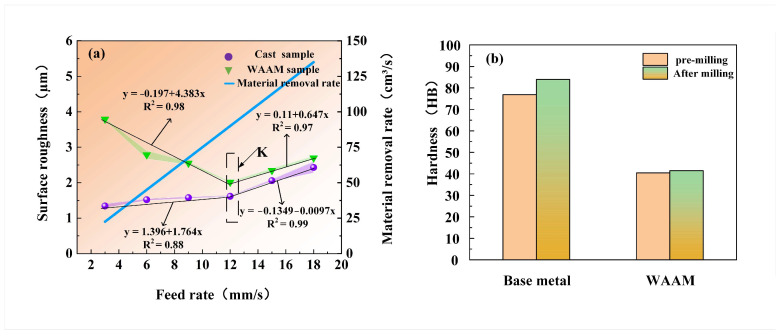
Feed rate experiment, (**a**) the relationship between feed rate and surface roughness, (**b**) hardness of point K in (**a**).

The relationship between feed rate and surface roughness is shown in Figure 7a. In the results, it is observed that the surface roughness of the as-cast sample increases with the increase in feed rate, ranging from 1.341 μm to 2.523 μm. When the feed rate is within the range of 2.5 mm/s to 12 mm/s, the rate of increase in surface roughness is relatively slow; when the feed rate exceeds 12 mm/s, the growth rate of surface roughness accelerates significantly. On the contrary, the surface roughness of the as-deposited sample shows a different trend with the variation in feed rate, ranging from 2.013 μm to 3.791 μm. Within the feed rate range of 2.5 mm/s to 12 mm/s, the surface roughness decreases rapidly with the increase in feed rate; however, when the feed rate exceeds 12 mm/s, the surface roughness begins to increase with the increase in feed rate. Notably, when the feed rate reaches 12 mm/s, the as-deposited sample exhibits the best surface quality.

To investigate the impact of feed rate on material hardness, samples under the inflection point K (spindle speed of 4500 r/min, feed rate of 12 mm/s, and cutting depth of 0.75 mm) were selected for hardness analysis. The results show that the hardness of the as-cast sample before milling is 76.901 HB, which increases to 86.433 HB after milling, while the hardness of the as-deposited sample before milling is 40.752 HB, and it increases slightly to 41.350 HB after milling, as shown in Figure 7b. This indicates that, under these parameters, the surface hardening of the as-cast sample is more significant after milling while the surface hardening of the as-deposited sample is relatively weaker.

**Figure 8:**
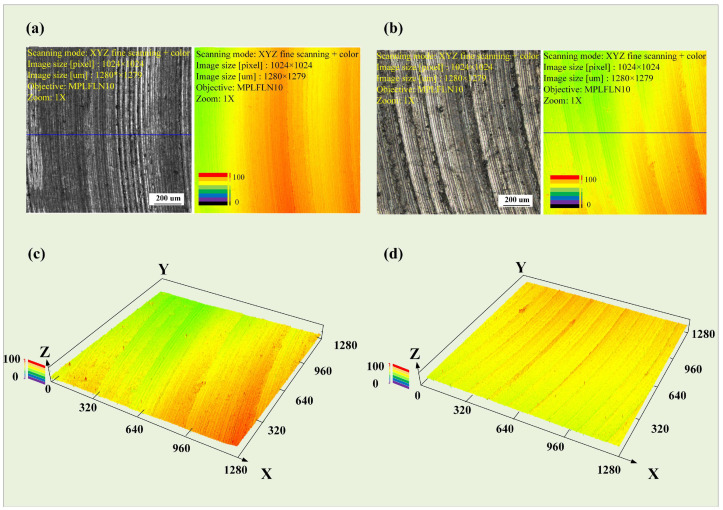
Feed rate experiment, (**a**) surface of as-cast sample, (**b**) surface of as-deposited sample, (**c**) 3D morphology of as-cast sample, (**d**) 3D morphology of as-deposited sample.

**Figure 9:**
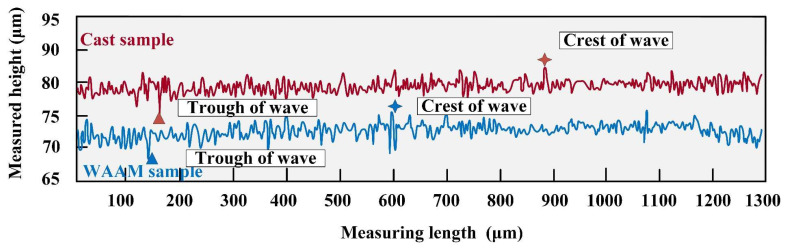
Surface contours of K point.

The two-dimensional surface morphologies of the as-cast and as-deposited samples at point K are shown in Figure 8a,b, respectively, while the three-dimensional morphologies are presented in Figure 8c,d, respectively. The results indicate that, under these parameters, the tool marks on the as-deposited sample are more pronounced, whereas the surface of the as-cast sample has a greater degree of undulation. The difference between the peak and valley of the as-cast sample is 7.503 μm, and the difference between the peak and valley of the as-deposited sample is 8.516 μm. Under these experimental parameters, the tool marks on both samples are not significant, and the processed surfaces are relatively smooth. However, the surface undulation of the as-cast sample is still slightly greater than that of the as-deposited sample. The results are shown in Figure 9.

### 3.3. Cutting Depth

**Figure 10:**
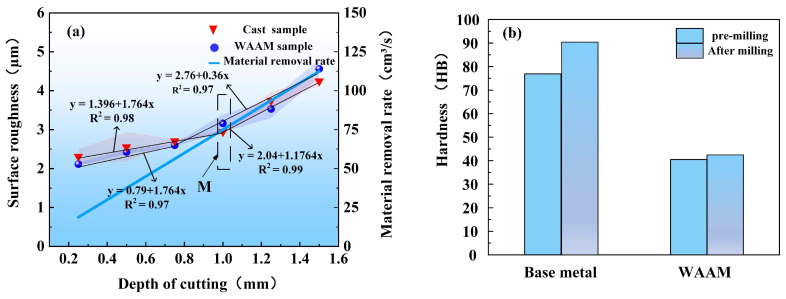
Cutting depth experiment, (**a**) the relationship between cutting depth and surface roughness, (**b**) hardness of point M in (**a**).

The relationship between cutting depth and surface roughness is shown in Figure 10a. For both as-cast and as-deposited samples, the surface roughness increases with the increase in cutting depth. The surface roughness of as-cast samples varies between 2.420 μm and 4.562 μm, while the range for as-deposited samples is between 2.311 μm and 4.310 μm. As the cutting depth increases, the surface roughness of as-cast samples exhibits a significant upward trend. Especially when the cutting depth exceeds 0.75 mm, the deterioration in surface quality becomes particularly severe. Based on this, this study selects samples at the inflection point M under specific parameters (spindle speed of 4500 r/min, feed rate of 7.5 mm/s, and cutting depth of 0.75 mm) for hardness analysis. The results indicate that the original hardness of as-cast samples is 76.906 HB, which increases to 88.603 HB after milling. For as-deposited samples, the original hardness is 40.751 HB, and it slightly increases to 41.051 HB after milling, as shown in Figure 10b.

**Figure 11:**
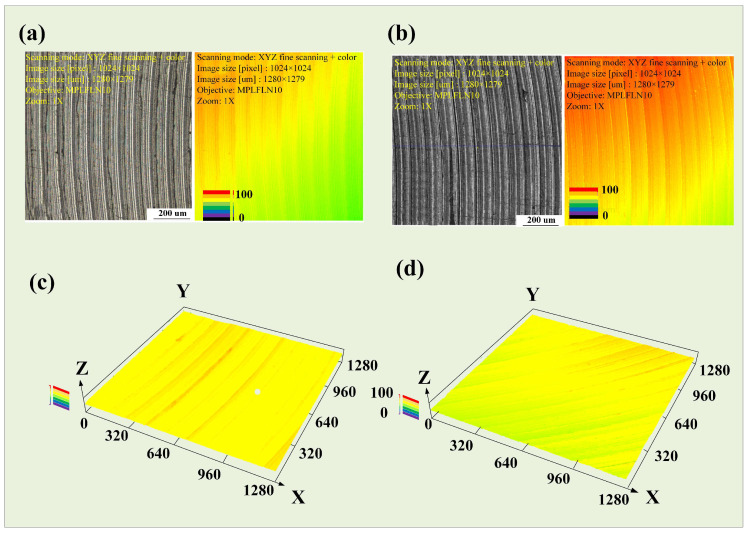
Cutting depth experiment, (**a**) surface of as-cast sample, (**b**) surface of as-deposited sample, (**c**) 3D mor phology of as-cast sample, (**d**) 3D morphology of as-deposited sample.

**Figure 12:**
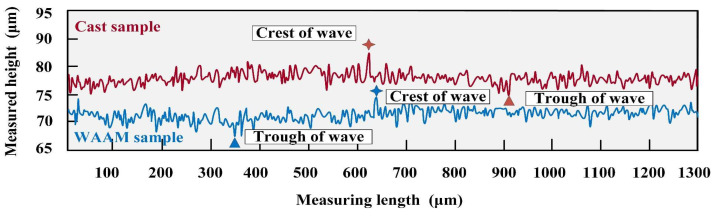
Surface contours of M point.

Under these parameters, although there are slight tool marks on the processed surface, it is still relatively smooth overall. The two-dimensional surface morphologies of as-cast and as-deposited samples are shown in Figure 11a,b, respectively, while their three-dimensional morphologies are presented in Figure 11c,d, respectively. The difference between the peak and valley in the as-cast sample is 10.265 μm, while the difference in the as-deposited sample is 7.518 μm. Under the current parameters, the tool marks on both samples are not significant, and the overall processed surface is relatively smooth, as shown in Figure 12.

### 3.4. Multiple Factors

Based on the measured results shown in Table 3, a second-order regression model relating surface roughness to spindle speed, feed rate, and cutting depth was obtained using the Design-Expert software. Subsequently, an ANOVA analysis was conducted.

As-cast:

(2) $$\mathrm{Ra}=2.82-0.95\times A+0.54\times B+2.05\times C-0.038\times \mathrm{AB}+0.37\times \mathrm{AC}+0.094\times \mathrm{CB}+1.03\times A^{2}+0.48\times B^{2}+1.59\times C^{2}$$

As-deposited:

(3) $$\mathrm{Ra}=3.48+0.18\times A+0.46\times B+0.036\times C+0.064\times \mathrm{AB}+0.093\times \mathrm{AC}+0.21\times \mathrm{BC}+0.51\times A^{2}-0.19\times B^{2}-0.45\times C^{2}-0.43\times A^{2}B-0.14\times A^{2}C-0.68\times AB^{2}$$

It can be seen from Table 4, the coefficient of determination R^2^ for the response model of the as-cast sample is 0.9705, indicating that the model can account for 97.05% of the variation in the response. The correlation coefficient is close to 1, suggesting a good fit of the model. According to the analysis of variance (with a significance level of α = 0.05, corresponding to a confidence interval of 0.95), the model has an F-value of 25.59 and a *p*-value of 0.0002, which is significantly smaller than 0.05, indicating a high level of significance. Based on the *p*-value, the rotational speed A and the cutting depth C have a significant impact on the surface quality. For the as-deposited sample, the coefficient of determination R^2^ for the response model is 0.9753, suggesting that the model can explain 97.53% of the variation in the response. The correlation coefficient is close to 1, indicating a good fit of the model. The analysis of variance (with a significance level of α = 0.05, corresponding to a confidence interval of 0.95) reveals an F-value of 15.57 and a *p*-value of 0.0084, which is significantly smaller than 0.05. Therefore, the model demonstrates a high level of significance. Based on the *p*-value, the influence of the three parameters can be ranked as follows: feed rate > rotational speed > cutting depth.

**Figure 13:**
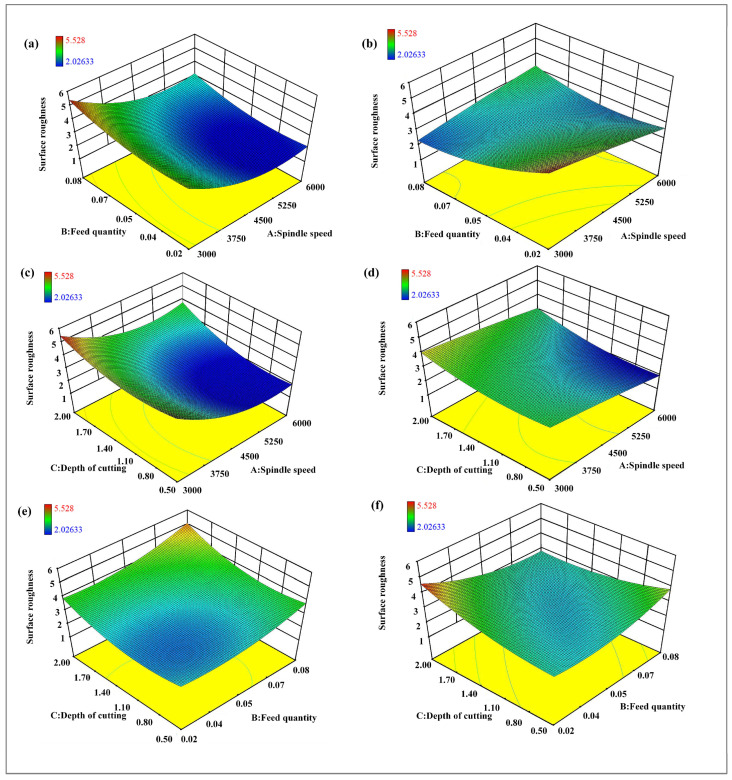
Response surface, (**a**,**b**) the interaction between spindle speed and feed rate in as-cast and as-deposited sample, (**c**,**d**) the interaction between spindle speed and cutting depth in as-cast and as-deposited sample, (**e**,**f**) the interaction between feed rate and cutting depth in as-cast and as-deposited sample.

The surface roughness of as-cast samples ranges from 2.011 μm to 5.512 μm, while the surface quality of as-deposited samples fluctuates between 2.203 μm and 4.928 μm. When adjusting different milling parameters, the milling surface quality of as-deposited samples exhibits relatively stable characteristics. Through the study of multi-factor interactions, as shown in Figure 13, it is observed that the surface roughness of as-cast samples gradually increases as the spindle speed decreases and the feed rate increases. Conversely, when the spindle speed reaches its peak and the feed rate is adjusted to its minimum, the surface quality of as-cast samples achieves the optimal state. For as-deposited samples, the surface roughness tends to increase as the spindle speed decreases and the feed rate decreases. The comparison indicates that there is a significant difference in the impact of feed rate on the surface roughness between the two types of samples.

The interaction between cutting depth and spindle speed has been investigated. For as-cast samples, the surface roughness increases as the cutting depth increases and the spindle speed decreases. However, in as-deposited samples, the surface roughness is relatively smooth, and the contour lines are not densely distributed, which suggests that the impact of cutting depth and spindle speed on the surface roughness of additive manufactured samples is not significant.

Regarding the interaction between cutting depth and feed rate, it is found that the surface roughness of as-cast samples increases as both parameters increase. Especially under the conditions of minimum feed rate and shallowest cutting depth, the surface quality of as-cast samples is the best. As-deposited samples exhibit similarities to as-cast samples in this aspect, but with a relatively weaker response.

## 4. Discussion

Significant differences exist in surface roughness between as-cast and as-deposited samples under milling parameters. In terms of spindle speed, as the spindle speed increases, the surface quality of both sample types exhibits an improving trend. This phenomenon can be attributed to the increased number of cutting actions per unit time due to the higher speed, which enhances metal cutting efficiency and improves surface roughness precision. When the spindle speed is too low, continuous chips are easily formed, leading to reduced surface quality. Simultaneously, the low speed reduces the generation of cutting heat, which helps to mitigate plastic deformation and thermal influence of the material, benefiting surface quality improvement [20]. As the speed further increases, the decreasing trend in surface roughness gradually slows down. The reason is that the elevated temperature in the cutting area leads to local softening of the metal, affecting material flow and hardness [21,22,23]. Although cutting forces decrease and vibrations and deformations reduce, the high viscosity of the material may cause chips to adhere to the cutting tool, resulting in surface damage. At this point, the negative impact of viscosity outweighs the positive impact of reduced hardness [24]. When the spindle speed increases to 6000 r/min, although the surface becomes smoother, the reduction in roughness tends to be minimal.

Due to the lower hardness of as-deposited samples, which is 43% lower than that of as-cast samples, when the spindle speed is low, the milling process generates smaller cutting forces and less elastic–plastic deformation, leading to a significantly better surface quality of as-deposited samples under the same milling parameters compared to as-cast samples [25]. In milling processes, surface morphology and roughness are primarily influenced by two factors: one is the scallop height caused by the rake angle of the cutting edge and the other is the plastic deformation of the material due to extrusion and recovery by the cutting edge. The wall morphology and roughness are mainly determined by the scallop height between cylindrical surfaces formed by tool feed and material plastic deformation. Differences in force during processing can lead to variations in surface quality. Due to the lower hardness and higher plasticity of as-deposited samples, milling can easily produce a built-up edge (BUE), resulting in a poorer surface roughness of the workpiece. As the spindle speed increases, the tendency for BUE formation is eliminated, improving surface quality. However, with further increases in spindle speed, the heat generated per unit time increases, raising the milling temperature in the deformation zone, leading to softening of the chip’s bottom layer and forming an extremely thin micro-melted layer [26], which reduces the friction coefficient on the rake face, thus showing a trend of decreasing surface roughness. But, when the spindle speed increases to a high level, the excessive heat generated during milling leads to excessively high milling temperatures, making it difficult for the heat accumulated on the contact surface between the tool and the workpiece to dissipate quickly [27]. The adhesion between the chip and the milling cutter, as well as the processed surface, increases, causing the chip to melt and adhere, resulting in the phenomenon of a BUE. This increases the milling force. As the spindle speed rises, the frequency of this phenomenon increases, and the negative impact caused by changes in material viscosity dominates. Therefore, when the spindle speed exceeds 4500 r/min, the processed surface begins to deteriorate. For as-deposited materials, this critical speed value has not been reached. Although both material states experience softening due to local temperature increases during milling, the aluminum alloy undergoes mechanical stress during processing, leading to work hardening of the processed surface after cooling, as shown in Figure 4b. Work hardening occurs when the metal is plastically deformed, and the crystal structure becomes denser, increasing hardness in some areas.

For as-deposited samples, when the feed rate increases from 3 mm/s to 12 mm/s, the surface roughness of the samples decreases from 3.791 μm to 2.013 μm, a reduction of 46.8%, showing a trend opposite to that of as-cast samples. In arc additive manufacturing, the heating area of the ion arc column is large, and the energy is relatively dispersed. After the material cools down, certain residual stresses are generated. High residual stresses can cause micro-cracks in the material to propagate, forming cutting zones and cutting marks during milling, leading to increased surface roughness. When the feed rate is low, it can cause excessive cutting, which is unfavorable for the discharge of cutting heat with the chip, resulting in poorer surface quality [28,29]. However, when the feed rate increases, the amount of material removed per tooth increases, and the chip thickness increases. Within a certain range, thicker chips can more effectively remove heat from the cutting area, reducing the size of the thermally affected zone. Additionally, thicker chips can also reduce friction between the tool and the processed surface, thereby reducing the likelihood of scratching and burning. At this point, the positive effect of thermal influence factors outweighs the negative effect of increased cutting forces, so the surface roughness of the workpiece decreases and the surface quality improves significantly. When the feed rate exceeds 12 mm/s, the surface roughness of the part begins to increase. This is because, as the cutting thickness increases, the cutting force increases sharply, generating a large amount of cutting heat, which raises the temperature in the cutting area, reduces material viscosity, and leads to poorer surface quality. Furthermore, differences in hardness, density, and material ductility between as-deposited and as-cast samples lead to different results.

Regarding the cutting depth, during the milling process, the surface roughness of the as-cast sample is 2.102 μm when the cutting depth is 0.25 mm. As the cutting depth increases to 1.5 mm, the surface roughness rises to 4.613 μm, resulting in a 115.8% deterioration in surface quality for the as-cast sample and a 112% deterioration for the as-deposited sample. This is because the cross-sectional area of the material shear zone increases with the cutting depth, leading to an increase in the material removal rate per unit time. This, in turn, causes a sharp increase in milling force, resulting in an increase in adhered particles and scratches on the surface and a deterioration in surface quality. When the cutting depth of the as-deposited sample is less than 1 mm, the axial cutting force varies less, resulting in better surface quality. Furthermore, an increase in cutting depth also leads to an accumulation of frictional heat during the cutting process, which raises the cutting temperature. High temperatures can cause plastic deformation of the material and the diffusion of thermally affected areas, further impacting the surface quality [30].

## 5. Conclusions

This paper investigates the impact of single-factor and multi-factor interactions on surface roughness through the design of milling experiments on deposited samples, and compares the results with those obtained from traditional as-cast samples.

1. In single-factor experiments, the surface roughness of as-cast samples decreases as the spindle speed increases. Conversely, the surface roughness of as-deposited samples initially decreases and then increases with an increase in spindle speed. The surface roughness of as-cast samples increases with an increase in feed rate while the trend for as-deposited samples is opposite to that of cast samples (within the speed range of 12 mm/s).
2. The surface roughness of both as-cast and as-deposited samples increases with an increase in cutting depth. The hardness of as-deposited samples is lower than that of as-cast samples, and both undergo a certain degree of surface hardening after milling.
3. In the analysis of multi-factor results, the surface roughness of as-cast samples is primarily influenced by spindle speed and cutting depth while the feed rate has a less significant impact on their surface quality. The optimal surface quality, with a roughness of 2.026 μm, is achieved at a spindle speed of 6000 r/min, a feed rate of 0.2 mm/r, and a cutting depth of 0.5 mm. The surface roughness of as-deposited samples is mainly affected by the feed rate, and the optimal surface quality is obtained at a spindle speed of 4500 r/min, a feed rate of 0.08 mm/r, and a cutting depth of 0.5 mm.

## Figures and Tables

**Table 1:** Results in different directions.

Direction	Ra_1_	Ra_2_	Ra_3_	Average	Surface Treatment
X+	2.738	2.792	2.962	2.831	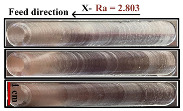
X-	2.752	2.799	2.861	2.803	
Y+	2.430	2.253	1.860	2.182	
Y-	2.031	2.182	2.321	2.177	
Processing Parameters					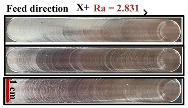
Factors	Level 1	Level 2	Level 3		
n (r/min)	4500	-	-		
v (mm/s)	-	7.5	-		
ap (mm)	-	-	0.5		
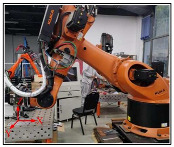					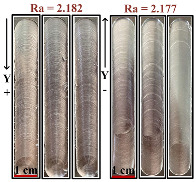

**Table 2:** Experimental design for single factor.

Specimens No.	Process Drawing	Milling Conditions (Factors)				
		a_p_	n	V	Ra	
	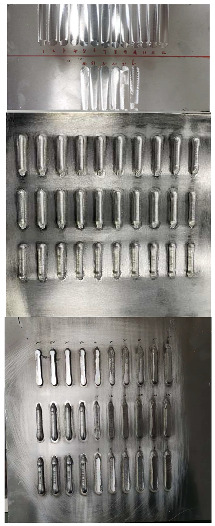				As-cast	As-deposition
Sp. 1		▲	1000	★	5.305	3.752
Sp. 2		▲	1500	★	5.082	3.614
Sp. 3		▲	2000	★	4.207	3.520
Sp. 4		▲	2500	★	3.624	3.181
		▲	3000	★	3.383	3.033
		▲	3500	★	3.068	2.518
		▲	4000	★	2.783	2.456
		▲	4500	★	2.325	2.152
		▲	5000	★	2.095	2.145
		▲	5500	★	1.683	2.341
		▲	6000	★	1.527	2.463
		▲	●	3	1.343	3.790
		▲	●	6	1.519	2.786
		▲	●	9	1.576	2.549
		▲	●	12	1.616	2.013
		▲	●	15	2.058	2.508
		▲	●	18	2.529	2.552
		0.25	●	★	2.113	2.026
		0.5	●	★	2.422	2.318
		0.75	●	★	2.598	2.707
		1	●	★	3.160	2.945
		1.25	●	★	3.530	3.813
Sp. 23		1.5	●	★	4.56	4.303
Factors			Level 1 ●		Level 2★	Level 3▲
n (r/min)			4500		-	-
v (mm/s)			-		7.5	-
ap (mm)			-		-	0.75

**Table 3:** Experimental design for multi-factors.

Specimens No.	Cutting Area	Milling Diagram		Milling Conditions (Factors)		
				n	f	a_p_
Sp. 1	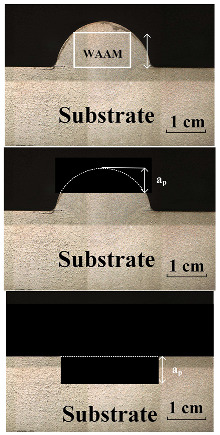	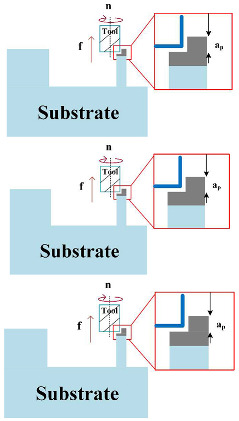		★	★	◆
Sp. 2				★	★	◆
Sp. 3				◆	★	▲
Sp. 4				◆	★	★
				◆	◆	◆
				◆	◆	◆
				◆	◆	◆
				▲	◆	▲
				★	◆	▲
				★	◆	★
				◆	◆	◆
				▲	◆	★
				◆	◆	◆
				◆	▲	★
				◆	◆	◆
				▲	◆	◆
Sp. 17				◆	◆	▲
Factors			Level 1 ★	Level 2◆		Level 3▲
n (r/min)			3000	4500		6000
f (mm/s)			0.02	0.05		0.08
a_p_ (mm)			0.5	1		1.5

★ indicates the first level of spindle speed is 4500 r/min, ◆ indicates the second level of feed speed is 7.5 mm/s, ▲ indicates the third level of cutting depth is 0.75 mm.

**Table 4:** ANOVA results.

Regression Model 1 (Casting)			Regression Model 2 (WAAM)		
Source	F-Value	*p*-Value	Source	F-Value	*p*-Value
A- Spindle speed	45.66	0.0003	A- Spindle speed	4.33	0.1060
B- Feed rate	3.28	0.1131	B- Feed rate	27.4	0.0064
C- Cutting depth	26.07	0.0014	C- Cutting depth	0.17	0.7053
AB	0.028	0.8726	AB	52.71	0.0019
AC	4.35	0.0754	AC	1.12	0.3495
BC	0.084	0.7801	BC	5.53	0.0783
A2	41.50	0.0004	A2	34.98	0.0041
B2	3.38	0.1088	B2	4.96	0.0900
C2	11.02	0.0128	C2	27.55	0.0063
Model	25.59	0.0002	Model	15.75	0.0084
R^2^	0.9705		R^2^	97.93	

## Data Availability

The original contributions presented in the study are included in the article, further inquiries can be directed to the corresponding author.
